# Risk adapted therapy for newly diagnosed multiple myeloma delivered through local cytogenetic laboratories in a National Clinical Trial: UKMRA RADAR study

**DOI:** 10.1002/jha2.1015

**Published:** 2025-06-09

**Authors:** Dipal Mehta, Robert Cicero, Laura Chiecchio, Samir Asher, Martin Kaiser, Sarah Gooding, Kara‐Louise Royle, Catherine Olivier, Doina Levinte, Charlotte Kennaway, Karthik Ramasamy, Kwee Yong, Matthew Jenner

**Affiliations:** ^1^ UCL Cancer Institute University College London London London UK; ^2^ Leeds Cancer Research UK CTU University of Leeds Leeds UK; ^3^ Wessex Genomics Laboratory Service (Salisbury) University Hospital Southampton Southampton UK; ^4^ Centre for Myeloma Research The Institute of Cancer Research, London, UK; ^5^ Department of Haematology MRC Molecular Haematology Unit Weatherall Institute of Molecular Medicine University of Oxford Oxford University Hospitals NHS Foundation Trust Oxford UK; ^6^ Radcliffe Department of Medicine Oxford UK; ^7^ Department of Haematology University Hospital Southampton Southampton UK

**Keywords:** cytogenetics, multiple myeloma, risk stratification

## Abstract

The UKMRA RADAR study is a phase II/III clinical trial for newly diagnosed multiple myeloma patients eligible for autologous stem cell transplant. It offers a risk‐adapted approach with the addition of isatuximab for genetically high‐risk patients, evaluated using local cytogenetics laboratories rather than centralised testing. We have observed excellent overall success rates, with > 90% patients assigned to a risk‐adapted pathway following cytogenetic testing in 25 local laboratories nationwide, with clinically‐relevant turnaround times allowing > 70% patients to commence isatuximab at the earliest opportunity if indicated. This paves the way for providing standard‐of‐care risk‐adapted treatment for multiple myeloma patients in the UK.

## 1 INTRODUCTION

The UKMRA RADAR study is an ongoing national, multi‐centre, risk‐adapted, response‐guided multi‐arm, multi‐stage (MAMS) phase II/III trial for patients with newly diagnosed multiple myeloma (MM) eligible for autologous stem cell transplant (ASCT). Recent studies in genetically‐defined high risk (HR) MM have reported high response rates with intensive induction and post‐ASCT consolidation and maintenance strategies, providing rationale for the use of a stratified and intensified treatment approach based on cytogenetic (CGN) risk [1, 2, 3, 4]. To facilitate a treatment strategy based on risk assignment, the ability to obtain accurate, reliable, and timely CGN results is crucial. This can be achieved efficiently through centralised testing in the context of a clinical trial, offering ready uniformity and quality control, as demonstrated by numerous frontline studies in MM [1, 5‐6]. However, since risk‐adapted treatment strategies in MM are likely to become standard‐of‐care, the use of local CGN laboratories is critical for genetic testing to be made available for all patients. As a transformative aim, we designed the UKMRA RADAR trial to utilize local accredited CGN laboratories. Here, we provide an update on this novel approach to MM risk‐stratification in the UK, demonstrating the feasibility of local testing in guiding risk‐adapted treatment approaches.

## 2 METHODS

CGN testing is conducted locally through interphase fluorescence in situ hybridization (FISH) or equivalent on CD138‐selected bone marrow cells, originating from the diagnostic or trial registration bone marrow aspirate (BMA). HR status is assigned centrally, following the presence of 2 or more of *t*(4;14), *t*(14;16), *t*(14:20), del(17p), del(1p32), and gain(1q). When the material available has not allowed exclusion of all relevant HR lesions, repeat CGN testing, or repeat BMA, is mandated. When risk assignment is not possible despite two BMAs, the patient is allocated to the standard risk pathway. Occasionally, risk stratification is undertaken despite only partial results if the unavailable results would not alter risk status. While awaiting risk stratification, all patients commence induction treatment with 4 cycles of RCyBorD (lenalidomide, cyclophosphamide, bortezomib, dexamethasone), with the addition of isatuximab from the second or third cycle for patients assigned HR, allowing ample time for patients who require repeat testing.

As local CGN laboratories across the United Kingdom (UK) employ differing testing algorithms and report templates, risk assignment is overseen by a genomics working group (GWG) composed of myeloma clinicians and cytogeneticists, using a scoring sheet to ensure a standardized approach (Figure S1). There is a target response time between receipt of CGN results and risk stratification of 48 h. Testing must be conducted on CD138‐selected BMA cells to increase the proportion of plasma cells for analysis [7]. Participating sites are briefed to use a ‘first pull’ BMA to reduce sampling artefact and to allow maximal yield of cells, in line with the British Society for Haematology guidance for newly diagnosed MM [8]. The precise method of CD138 enrichment and thresholds for classifying failed purification are left to the discretion of individual CGN laboratories

Agreed thresholds to signify clinically‐relevant results include a clone size > 10% for primary IgH translocations, and > 20% for copy number changes, as recommended by the European Myeloma Network (EMN) [9]. The overall plasma cell purity post CD138‐seperation is considered, as this directly affects the size of the abnormal clone/s. As smaller subclones may still be prognostically relevant, cases with abnormality levels below the agreed thresholds are discussed on a case‐by‐case basis within the wider GWG to establish a consensus.

## 3 RESULTS

As of February 1, 2024, the trial has recruited 720 patients from 72 sites across the UK. Of the 720 patients, 39 (5.4%) are still in the risk stratification process. Therefore, a total of 681 have completed risk stratification as per protocol and are reported on here. The median age is 61 years, with 58.4% males, 84.4% white, and 15.6% other ethnicities (Table S1).

Twenty‐five local laboratories across the UK have been used for CGN testing. Of the 681 patients, 72.7% (495/681) were assigned standard risk, 17.5% (119/681) HR, and 9.8% (67/681) were unable to be stratified and treated as standard risk. The prevalence of various HR cytogenetic abnormalities is summarized in Table 1.

**TABLE 1:** Genetic risk markers.

Genetic risk markers				
	Standard‐risk (*N* = 495)	High‐risk (*N* = 119)	Unable to determine (*N* = 67)	Total (*N* = 681)
***t*(4;14)**				
Detected	16 (3.2%)	53 (44.5%)	0 (0.0%)	69 (10.1%)
Not detected	469 (94.7%)	62 (52.1%)	30 (44.8%)	561 (82.4%)
Unable to determine	0 (0.0%)	2 (1.7%)	26 (38.8%)	28 (4.1%)
Not tested	10 (2.0%)	2 (1.7%)	11 (16.4%)	23 (3.4%)
***t*(14;16)**				
Detected	4 (0.8%)	18 (15.1%)	0 (0.0%)	22 (3.2%)
Not detected	471 (95.2%)	96 (80.7%)	25 (37.3%)	592 (86.9%)
Unable to determine	0 (0.0%)	1 (0.8%)	25 (37.3%)	26 (3.8%)
Not tested	20 (4.0%)	4 (3.4%)	17 (25.4%)	41 (6.0%)
***t*(14;20)**				
Detected	0 (0.0%)	6 (5.0%)	0 (0.0%)	6 (0.9%)
Not detected	430 (86.9%)	102 (85.7%)	4 (6.0%)	536 (78.7%)
Unable to determine	0 (0.0%)	1 (0.8%)	24 (35.8%)	25 (3.7%)
Not tested	65 (13.1%)	10 (8.4%)	39 (58.2%)	114 (16.7%)
**del(17p)**				
Detected	26 (5.3%)	34 (28.6%)	6 (9.0%)	66 (9.7%)
Not detected	468 (94.5%)	83 (69.7%)	30 (44.8%)	581 (85.3%)
Unable to determine	0 (0.0%)	0 (0.0%)	23 (34.3%)	23 (3.4%)
Not tested	1 (0.2%)	2 (1.7%)	8 (11.9%)	11 (1.6%)
**Gain(1q)**				
Detected	110 (22.2%)	109 (91.6%)	22 (32.8%)	241 (35.4%)
Not detected	385 (77.8%)	10 (8.4%)	7 (10.4%)	402 (59.0%)
Unable to determine	0 (0.0%)	0 (0.0%)	26 (38.8%)	26 (3.8%)
Not tested	0 (0.0%)	0 (0.0%)	12 (17.9%)	12 (1.8%)
**del(1p)**				
Detected	23 (4.6%)	39 (32.8%)	4 (6.0%)	66 (9.7%)
Not detected	444 (89.7%)	71 (59.7%)	21 (31.3%)	536 (78.7%)
Unable to determine	1 (0.2%)	0 (0.0%)	25 (37.3%)	26 (3.8%)
Not tested	27 (5.5%)	9 (7.6%)	17 (25.4%)	53 (7.8%)
***t*(11;14)**				
Detected	77 (15.6%)	6 (5.0%)	1 (1.5%)	84 (12.3%)
Not detected	354 (71.5%)	99 (83.2%)	16 (23.9%)	469 (68.9%)
Unable to determine	0 (0.0%)	1 (0.8%)	24 (35.8%)	25 (3.7%)
Not tested	64 (12.9%)	13 (10.9%)	26 (38.8%)	103 (15.1%)

**FIGURE 1:**
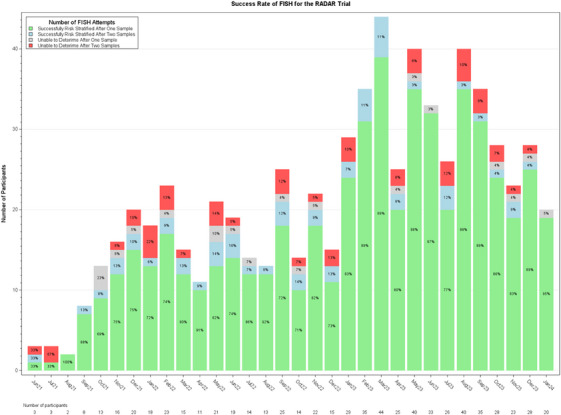
Success rate of cytogenetic risk stratification over time.

The rate of successful risk stratification following a first or second cytogenetic test is 90.2% (614/681). Risk has been successfully assigned from a first BM sample in 85.3% (581/681) of patients. 14.7% (100/681) of patients proceeded to a second BM sample; the success rate of repeat samples is 53% (53/103). Figure 1 shows the success rates of first and repeat tests over time.

Median turnaround time, measured as the number of days from day 1 of induction treatment until the study site is notified of the result, is 9 days (IQR −1 to 19). Sites were sent the outcome of genetic stratification prior to cycle 1 of induction treatment in 24.1% (164/681) patients, prior to cycle 2 in 70.8% (482/681) patients, and prior to cycle 3 in 83.0% (565/681) patients.

## 4 DISCUSSION

The RADAR study demonstrates that risk‐stratified treatment approaches are feasible in the context of a national phase II/III clinical trial using local CGN laboratories for risk assignment. The vast majority of patients have been allocated to a risk‐adapted treatment pathway following a first successful CGN test. HR status has been assigned in 17.5% of cases, which is consistent with previous literature on multi‐hit MM [10]. The most recent iteration of the study protocol requires time‐critical risk‐stratification for the addition of isatuximab to induction treatment from cycle 2 or cycle 3 in HR patients, and this has been shown to be achievable. We also demonstrate the advantage of retesting, with reasonably high success rates of repeat CGN tests ensuring that maximal numbers of patients are appropriately risk‐stratified.

CGN laboratories operating in the UK are accredited through GenQA (genomics quality assessment), which provides external quality assessments for the genomics clinical service. Regular independent assessments ensure the accuracy of the analytical results and provide a means for standardisation of myeloma CGN results across UK laboratories. Despite this, there remain several areas of heterogeneity between laboratories, which need to be considered. There is variability in sample acceptability criteria set by individual laboratories and whether FISH testing strategies are adapted based on the quality of the testing material. There are also differences with regards to the number of plasma cells analysed following CD138‐enrichment, which may affect the ability to detect low‐level abnormalities. The use of commercial FISH probes from different manufacturers which may have varying designs and coverage of genomic areas of interest, and by differing testing strategies between laboratories, adds to the heterogeneity of testing. We also report occasional CGN laboratories additionally employing multiplex ligation‐dependent probe amplification (MLPA), which has emerged as an effective method for detecting copy number variations with good concordance with FISH [11]. These points of variability are specifically considered during the risk stratification process, and repeat testing is required in cases where unsuitable CD138+ purity, inappropriate FISH probe combinations, and discrepant overall testing strategies compromise the final result. We have also developed an internal trial‐focused quality control process through monthly performance reviews, undertaken by the GWG, of individual CGN laboratories and hospital sites. This allows issues such as unsatisfactory testing strategies to be flagged, and for individual laboratories or hospital sites to be contacted. This central monitoring directed by the GWG enables a further means of maintaining consistency between laboratories and is strength of our current approach.

Overall, we report a novel approach in utilizing local CGN laboratories for risk assignment as part of a national clinical trial, which we will continue to implement within the ongoing UKMRA RADAR study. This paves the way to providing standard‐of‐care risk‐adapted treatment as is currently seen in acute myeloid leukaemia (AML) and chronic lymphocytic leukaemia (CLL). We have been able to gather insight into standard‐of‐care CGN testing for patients with MM across the UK, and to reflect how real‐world designation of risk status is currently obtained. Our study has allowed valuable learning at site level and further strengthening of the genomic service nationally, whilst helping to reduce trial costs. We have observed that CGN testing is offered through a network model whereby hospitals across the UK are linked to specific CGN laboratories, allowing a streamlined service with oversight from CGN experts. The network model enables a degree of standardisation standardization of testing, as well as clinically relevant turnaround times. This model is applicable internationally in both public and privately funded healthcare systems and supports the infrastructure for accessible genetic risk stratification for all newly diagnosed MM patients.

## AUTHOR CONTRIBUTIONS

Dipal Mehta wrote the paper. Dipal Mehta, Samir Asher, Matthew Jenner, Kwee Yong, Martin Kaiser, and Laura Chiecchio performed central cytogenetic assessment. Robert Cicero, Kara‐Louise Royle, Doina Levinte, Catherine Olivier, and Charlotte Kennaway analysed the data and provided study support. Matthew Jenner, Kwee Yong, Karthik Ramasamy, Dipal Mehta, Sarah Gooding, Martin Kaiser, Laura Chiecchio, Kara‐Louise Royle, and Robert Cicero critically reviewed the paper. Matthew Jenner, Kwee Yong, and Karthik Ramasamy designed the study.

## CONFLICT OF INTEREST STATEMENT

The authors declare no conflicts of interest.

## ETHICS APPROVAL

The RADAR trial is performed in accordance with the recommendations guiding physicians in biomedical research involving human subjects adopted by the 18th World Medical Assembly, Helsinki, Finland, 1964, amended at the 48th World Medical Association General Assembly, Somerset West, Republic of South Africa, October 1996. Informed written consent is obtained from the participants prior to registration into the trial. REC Reference: 20/LO/0238

## CLINICAL TRIAL REGISTRATION

ISRCTN Number: 46841867. NIHR CPMS ID: 44923. EudraCT Number: 2019‐001258‐25

## PATIENT CONSENT STATEMENT

The authors have confirmed patient consent statement is not needed for this submission.

## Supporting information



Supporting information

## Data Availability

The data that support the findings of this study are available on request from the corresponding author. The data are not publicly available due to privacy or ethical restrictions.
