# Supplementary material for: Risk adapted therapy for newly diagnosed multiple myeloma delivered through local cytogenetic laboratories in a National Clinical Trial: UKMRA RADAR study
Source: EJHaem. 2025 Jun 9;6(3):e1015. doi: 10.1002/jha2.1015 (PMC12172564; doi:10.1002/jha2.1015)
Supplement: Supplementary file 1 — Supporting information [file JHA2-6-e1015-s001.docx]

**Supplementary Data:**

**Table S1: Clinical characteristics at diagnosis**

|  | **Standard-risk**  **(N=495)** | **High-risk**  **(N=119)** | **Unable to determine**  **(N=67)** | **Total**  **(N=681)** |
| --- | --- | --- | --- | --- |
| **Baseline Characteristics** | | | | |
| **Age at Main Trial Registration (Years)** |  |  |  |  |
| Median (IQR) | 61.0 (55.0, 66.0) | 60.0 (54.0, 66.0) | 62.0 (56.0, 67.0) | 61.0 (55.0, 66.0) |
| **Participants Sex** |  |  |  |  |
| Male | 301 (60.8%) | 58 (48.7%) | 39 (58.2%) | 398 (58.4%) |
| Female | 194 (39.2%) | 61 (51.3%) | 28 (41.8%) | 283 (41.6%) |
| **Ethnicity** |  |  |  |  |
| White | 419 (84.6%) | 98 (82.4%) | 58 (86.6%) | 575 (84.4%) |
| Mixed / Multiple ethnic groups | 3 (0.6%) | 0 (0.0%) | 1 (1.5%) | 4 (0.6%) |
| Asian / Asian British | 17 (3.4%) | 3 (2.5%) | 2 (3.0%) | 22 (3.2%) |
| Black / Black British / Caribbean / African | 17 (3.4%) | 7 (5.9%) | 3 (4.5%) | 27 (4.0%) |
| Other ethnic group | 5 (1.0%) | 3 (2.5%) | 1 (1.5%) | 9 (1.3%) |
| Prefer not to state / Unknown | 34 (6.9%) | 8 (6.7%) | 2 (3.0%) | 44 (6.5%) |
| **ISS Stage** |  |  |  |  |
| Stage I | 190 (38.4%) | 34 (28.6%) | 23 (34.3%) | 247 (36.3%) |
| Stage II | 205 (41.4%) | 50 (42.0%) | 28 (41.8%) | 283 (41.6%) |
| Stage III | 74 (14.9%) | 31 (26.1%) | 15 (22.4%) | 120 (17.6%) |
| Missing | 26 (5.3%) | 4 (3.4%) | 1 (1.5%) | 31 (4.6%) |
| **R-ISS** |  |  |  |  |
| Stage 1 | 16 (3.2%) | 24 (20.2%) | 2 (3.0%) | 42 (6.2%) |
| Stage 2 | 411 (83.0%) | 73 (61.3%) | 22 (32.8%) | 506 (74.3%) |
| Stage 3 | 22 (4.4%) | 15 (12.6%) | 4 (6.0%) | 41 (6.0%) |
| Missing | 46 (9.3%) | 7 (5.9%) | 39 (58.2%) | 92 (13.5%) |
| **ECOG Status** |  |  |  |  |
| 0 | 254 (51.3%) | 62 (52.1%) | 42 (62.7%) | 358 (52.6%) |
| 1 | 164 (33.1%) | 37 (31.1%) | 19 (28.4%) | 220 (32.3%) |
| 2 | 45 (9.1%) | 13 (10.9%) | 4 (6.0%) | 62 (9.1%) |
| 3 | 11 (2.2%) | 3 (2.5%) | 2 (3.0%) | 16 (2.3%) |
| 4 | 2 (0.4%) | 0 (0.0%) | 0 (0.0%) | 2 (0.3%) |
| Missing | 19 (3.8%) | 4 (3.4%) | 0 (0.0%) | 23 (3.4%) |
| **Serum B2 microglobulin** |  |  |  |  |
| Median (IQR) | 3.3 (2.5, 4.7) | 3.8 (2.8, 5.6) | 3.5 (2.7, 5.3) | 3.3 (2.6, 4.9) |
| Missing | 26 | 4 | 1 | 31 |
| **Serum albumin** |  |  |  |  |
| Median (IQR) | 37.0 (32.0, 41.0) | 35.0 (31.0, 41.0) | 37.0 (30.0, 41.0) | 36.0 (32.0, 41.0) |
| Missing | 17 | 2 | 1 | 20 |
| **Lactate dehydrogenase (LDH)** |  |  |  |  |
| Median (IQR) | 176 (143, 227) | 190 (146, 243) | 193 (146, 246) | 179 (144, 232) |
| Missing | 25 | 7 | 1 | 33 |
| **Haemoglobin (Hb)** |  |  |  |  |
| Median (IQR) | 117 (103, 129) | 103 (90.0, 124) | 115 (103, 128) | 115 (101, 128) |
| Missing | 16 | 2 | 1 | 19 |
| **Serum creatinine** |  |  |  |  |
| Median (IQR) | 76.0 (65.0, 90.0) | 76.0 (64.0, 98.0) | 80.5 (67.0, 98.0) | 77.0 (65.0, 92.0) |
| Missing | 18 | 2 | 1 | 21 |
| **Corrected serum calcium** |  |  |  |  |
| Median (IQR) | 2.4 (2.3, 2.5) | 2.4 (2.3, 2.5) | 2.4 (2.4, 2.5) | 2.4 (2.3, 2.5) |
| Missing | 19 | 3 | 1 | 23 |
| **Paraprotein type** |  |  |  |  |
| IgG | 286 (57.8%) | 68 (57.1%) | 46 (68.7%) | 400 (58.7%) |
| IgA | 111 (22.4%) | 36 (30.3%) | 13 (19.4%) | 160 (23.5%) |
| IgM | 3 (0.6%) | 0 (0.0%) | 0 (0.0%) | 3 (0.4%) |
| IgD | 4 (0.8%) | 1 (0.8%) | 1 (1.5%) | 6 (0.9%) |
| Light Chain Only | 67 (13.5%) | 10 (8.4%) | 6 (9.0%) | 83 (12.2%) |
| Non-Secretory (no PP, LCs or urine BJP detected) | 6 (1.2%) | 1 (0.8%) | 1 (1.5%) | 8 (1.2%) |
| Missing | 18 (3.6%) | 3 (2.5%) | 0 (0.0%) | 21 (3.1%) |

**Figure S1: Genetic Risk Scoring Manual**

This document is to be used by the CI / delegate to determine the genetic risk status of participants entering the trial.

Contents

[Myeloma XV (RADAR): Definition of High-Risk 1](#_Toc175838706)

[Myeloma XV (RADAR): Sample requirements prior to assigning risk 1](#_Toc175838707)

[Myeloma XV (RADAR): Repeat Bone Marrow Sample Flow Diagram 2](#_Toc175838708)

[Myeloma XV (RADAR): Incomplete FISH Results Table 3](#_Toc175838709)

# Myeloma XV (RADAR): Definition of High-Risk

A participant is defined to be high-risk on the Myeloma XV (RADAR) trial if they have two or more of the following cytogenetic abnormalities detected.

- t(4;14)
- t(14;16)
- t(14;20)
- del(17p)
- gain(1q)
- del(1p)

If the results are inconclusive for any of the six high-risk cytogenetic abnormalities i.e. it is not clear whether the adverse marker is detected or not, then the following section should be used to determine if a repeat bone marrow sample is required.

# Myeloma XV (RADAR): Sample requirements prior to assigning risk

The following criteria must be met prior to confirming genetic risk status. Please discuss with a member of the genomics working group if risk status cannot be assigned.

- Cytogenetic analysis should be undertaken on CD138-selected bone marrow cells.
- Copy number changes should meet a 20% cut-off level to signify a positive FISH result.
  However, this is dependent on the plasma cell purity. If the plasma cell purity post CD138-separation is not given, but a primary IGH translocation is present, it is possible to adjust the clone size of the low-level copy number change based on the level of IGH-rearrangement.
- Translocations should meet a 10% cut-off level to signify a positive FISH result (regardless of probe used).
- If CD138 purity is reported, this should be considered to adjust the clone size as potentially low-level abnormalities (detected at <20%) could become significant prognostic markers
- Cases with borderline clone sizes should be discussed amongst GWG for a consensus decision
- Translocation probes (normally listed in each report):
- If the probe used is sensitive for any breakapart at the IgH locus and is negative, all translocations involving IgH can be excluded (most labs will use a breakapart probe)
- If the probe used is specific for one translocation only (dual fusion), a minimum of t(4;14) and t(14;16) must be tested.
- If t(14;20) is the only high risk abnormality missing, repeat testing is not required
- Not all labs will test for t(11;14). As this is a standard risk feature, this is not a mandatory test
- If all translocation testing fails, repeat testing may not be required if there are informative results for del(1p), gain(1q) and del(17p) and the case can be assigned to a specific risk category based on these markers
- If there is a gain(1q), consider whether there’s a background tetraploidy as this may not represent a true gain(1q)
- Gain(1q) is a poor prognostic marker regardless of the 1p status. Therefore, gain of chromosome 1 can be considered as gain(1q)
- Loss of chromosome 1 can be considered as del(1p)
- Loss of chromosome 17 centromere with no proven TP53 abnormalities does not represent a high risk marker; similarly, gain of chromosome 17 centromere with normal TP53 does not constitute loss of TP53
- Hyperdiploidy is often not tested for unless MLPA or conventional cytogenetics are used. A FISH method that can define hyperdiploidy requires gains of signals for any 2 of the chromosomes 5, 9 or 15.

If a laboratory has reported an abnormality at a lower percentage another member of the working group should be contacted, and a consensus reached.

# Myeloma XV (RADAR): Repeat Bone Marrow Sample Flow Diagram

This flow diagram is to determine whether a repeat bone marrow sample should be requested when at least one of the six high-risk cytogenetic abnormalities have an inconclusive result, i.e. are not stated to be detected or not detected based on the first report.


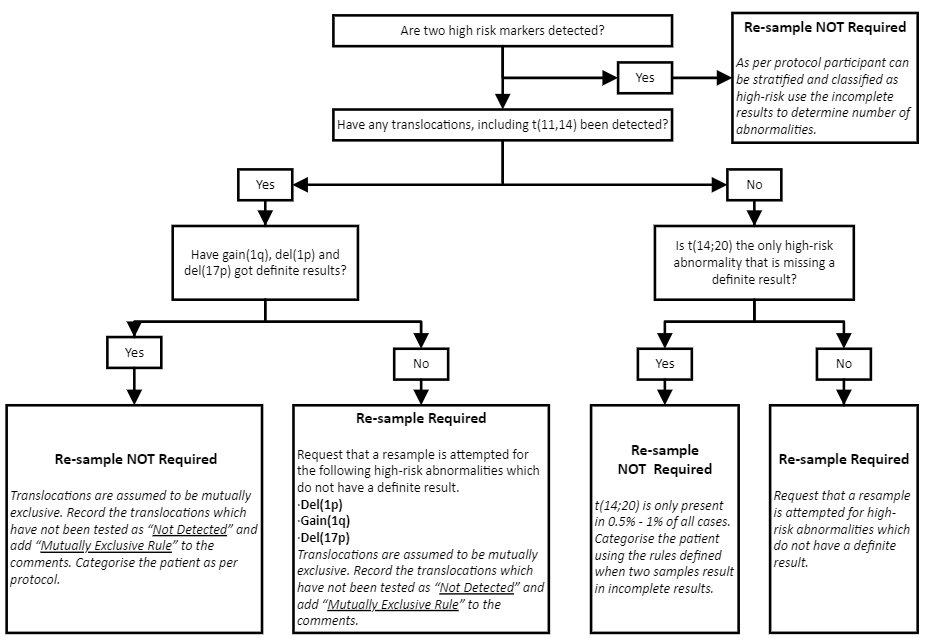


# Myeloma XV (RADAR): Incomplete FISH Results Table

This table is to determine how to categorise a patient into a genetic risk category when, after two (where applicable) attempted bone marrow samples, the final results are still incomplete. I.e. at least one of the six high-risk cytogenetic abnormalities has an inconclusive result and is not stated to be ‘detected’ or ‘not detected’.

Note this table refers to the number of high-risk abnormalities which have been **detected** after analysis of both (where applicable) samples.

| **Number of High-Risk Abnormalities Detected** | **Genetic Risk Pathway** | **Possible Randomisation** | **Category for the number of high-risk adverse lesions at trial registrations stratification factor** |
| --- | --- | --- | --- |
| 0 | Standard Risk | R1 or R2* | Unable To Determine |
| 1 | Standard Risk | R1 or R2* | Unable To Determine |
| 2 | High-Risk | N/A** | Unable To Determine** |
| 3 | High-Risk | N/A** | 3 or More** |
| 4 | High-Risk | N/A** | 3 or More** |
| 5 | High-Risk | N/A** | 3 or More** |

**Dependent on MRD status at 100 days post-ASCT*

*** This scoring manual is for participants registered after V4 of the protocol where randomisation 3 was removed.*

It is important to remember that translocations (t(4;14), t(14;16), t(14;20) & t(11,14)) are mutually exclusive so if one is detected it is not possible for the participant to have any of the other translocations. The high-risk markers del(17p), gain(1q) and del(1p) are not mutually exclusive, therefore it is possible to have one or more of these.

When counting number of high-risk abnormalities not tested:

- Only consider translocations as ‘not tested’, if no other translocation has been detected
- The maximum ‘number of abnormalities’ a participant can have when they have untested translocations is one.
- In cases where the lab have used an IGH breakapart probe to test for IGH rearrangements and the result is ‘not detected’; all translocations should be recorded as ‘not detected’.
- Note that the translocation t(11,14) is not considered one of the high-risk cytogenetic abnormalities as per the RADAR definition, therefore in the cases where this is tested and detected but the other translocations are not tested, the ‘number of abnormalities’ possible for the rest of the untested translocations will be 0.
